# Primary Cutaneous Cryptococcosis Treated with Debridement and Fluconazole Monotherapy in an Immunosuppressed Patient: A Case Report and Review of the Literature

**DOI:** 10.1155/2015/131356

**Published:** 2015-02-02

**Authors:** Jennifer Wang, Luther Bartelt, Deborah Yu, Anjali Joshi, Bradley Weinbaum, Tiffany Pierson, Michael Patrizio, Cirle A. Warren, Molly A. Hughes, Gerald Donowitz

**Affiliations:** ^1^School of Medicine, University of Virginia, Charlottesville, VA 22903, USA; ^2^Division of Infectious Diseases, Department of Internal Medicine, University of Virginia, Charlottesville, VA 22903, USA; ^3^Department of Plastic and Maxillofacial Surgery, University of Virginia, Charlottesville, VA 22903, USA; ^4^Department of Internal Medicine, University of Virginia, Charlottesville, VA 22903, USA

## Abstract

*Cryptococcus neoformans* is an opportunistic yeast present in the environment. Practitioners are familiar with the presentation and management of the most common manifestation of cryptococcal infection, meningoencephalitis, in patients with AIDS or other conditions of immunocompromise. There is less awareness, however, of uncommon presentations where experience rather than evidence guides therapy. We report a case of primary cutaneous cryptococcosis (PCC) in a patient who had been immunosuppressed by chronic high-dose corticosteroid for the treatment of severe asthma. This case highlights the importance of early recognition of aggressive cellulitis that fails standard empiric antibiotic treatment in an immunocompromised patient. It also demonstrates successful treatment of PCC with a multispecialty approach including local debridement and fluconazole monotherapy.

## 1. Introduction

*Cryptococcus* is an encapsulated yeast, classified into four serotypes based on immunologic reactivity of the capsular polysaccharides [1].* C. neoformans* var.* neoformans* belongs to serotype D and is the most prevalent serotype in the USA [2]. It is present in the environment and has been isolated from pigeon droppings, decaying wood, fruits, and vegetables [3]. Patients with impaired cell-mediated immunity, such as those infected with HIV, solid-organ transplant recipients, and those on chronic corticosteroid therapy, are most vulnerable to cryptococcal infections [4].* Cryptococcus* can present with a variety of skin and soft tissue manifestations including acneiform lesions, purpura, vesicles, nodules, abscesses, ulcers, granulomas, pustules, draining sinuses, and cellulitis. Most skin and soft tissue manifestations occur in the setting of disseminated disease, which is apparent in 10–15% of patients with systemic cryptococcosis [5]. Primary cutaneous infection as a result of direct local inoculation, however, is rare.

## 2. Case

**Figure 1:**
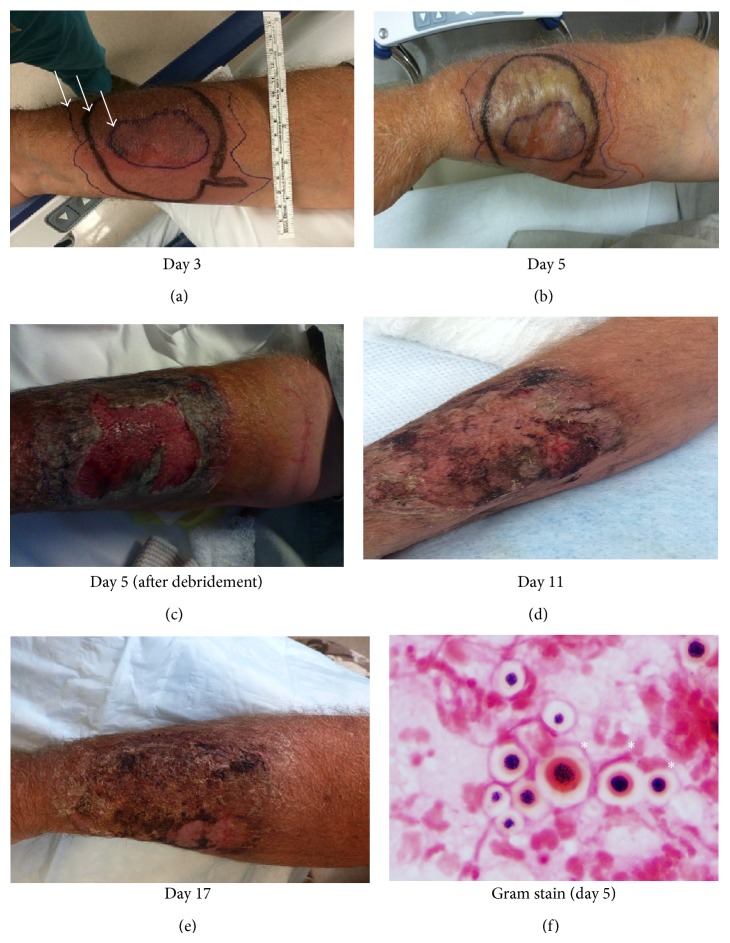
Primary cutaneous cryptococcosis of the right forearm. Progressive skin changes documented at the initial visit to the emergency department on the third day of symptoms (a), during hospitalization (b-c), and at postdischarge clinic follow-up (d). White arrows denote the outlines of the region of erythema marked on each respective day prior to presentation (i.e., Days 1–3). Fluconazole was initiated along with two interrupted doses of amphotericin on Day 3. Broad-spectrum antibacterial therapy with vancomycin and piperacillin-tazobactam (Day 5–Day 7), ampicillin-sulbactam (Day 8–Day 10), and amoxicillin-clavulanate (Day 11–Day 17) was also administered. (e) Wound improvement seen on one-week follow-up in clinic. The gram stain (f) from fluid expressed on Day 5 demonstrated abundant variably sized encapsulated spherical yeast forms (∗) that were identified as* C. neoformans* on blood agar.

A 56-year-old male carpenter with a past medical history of hypertension, uncontrolled diabetes mellitus, and severe asthma had been treated with prolonged courses of oral prednisone up to 100 milligrams (mg) daily for several months and at least 50 mg daily for the past 7 consecutive months. He presented to the emergency department (ED) with a rapidly enlarging plaque on his right forearm. Five days prior to admission, the patient was moving firewood in his backyard when he noticed a pustule on his volar right forearm. The pustule became erythematous and indurated throughout the next day, and the patient presented to a walk-in clinic where clindamycin was prescribed. Despite this therapy, the erythema continued to expand, and the forearm became painful. The patient represented to his primary care doctor who added linezolid to his antibiotic regimen. Of note, the patient was intolerant to penicillin, sulfa drugs, erythromycin, ciprofloxacin, doxycycline, and metronidazole. Despite the change in antibiotic therapy, the erythema continued to expand prompting a visit to the ED (Figure 1(a)). He denied fever, night sweats, malaise, or other systemic symptoms. A pustule was noted on exam with minimal fluid expressed. A bedside ultrasound was performed that did not reveal a subcutaneous abscess. The patient was sent home with the instruction to continue clindamycin and linezolid. The patient noticed worsening of his rash with purulent drainage and returned to the ED the next day. He was found to be afebrile with a pulse of 110 beats per minute, a respiratory rate of 20 breaths per minute, and blood pressure of 141/85 mmHg. Physical examination revealed a single 7 cm painful bulla with surrounding erythema that extended proximal to the elbow (Figure 1(b)). Lymphadenopathy was absent. Laboratory testing was significant for a leukocytosis of 17,500 cells/*μ*L (83.6% neutrophils, 1.8% band, and 1.8% atypical lymphocyte) and a hemoglobin A1c of 8.8%.

The antibiotics were changed to vancomycin and piperacillin/tazobactam, and the patient was admitted to the general medicine service. Plastic surgery was consulted. The epidermis of the bulla was carefully removed, draining a copious amount of purulent material with an unusual watery consistency. The expressed fluid gram stain revealed yeast forms and no bacteria (Figure 1(f)). Culture of the purulent material grew only* Cryptococcus neoformans*. Serological investigation for HIV was negative, and the patient's risk factor for immunosuppression was identified to be the chronic prednisone use. The patient was subsequently initiated on intravenous liposomal amphotericin B at 3 mg/kg/day for potential disseminated cryptococcal infection. However, he developed severe chest pain during liposomal amphotericin B administration that resolved after termination of the infusion. A second trial of liposomal amphotericin B was also terminated early due to recurrence of chest pain. In total, he received less than 300 mg (one dose) of liposomal amphotericin B. A decision was made to initiate oral fluconazole (12 mg/kg/day), which he tolerated well.

Workup for disseminated disease and meningeal involvement including serum cryptococcal antigen, blood culture, urine culture, computed tomography scan of the head, chest, abdomen, and pelvis, and cerebral spinal fluid (CSF) analysis for cryptococcal antigen all returned negative. The fluconazole dose was decreased to 8 mg/kg/day. Serial liver function test and QTc were monitored. In addition to systemic therapy for* Cryptococcus*, daily dressing changes with a Pluronic antimicrobial cream manufactured at our institution (Kolliphor P 188 50%, polymyxin 10,000 units/gram, nystatin 4,000 units/gram, and nitrofurantoin 0.3% within a Pluronic F68 carrier) were performed for topical preemptive antibacterial and antifungal coverage during wound healing. The patient continued to feel well with improvement in his arm tenderness. However, the surrounding erythema persisted with worsening leukocytosis that peaked at 22,000 cells/*μ*L, raising concern for a deeper infection that was not apparent on exam. An MRI of the arm was consistent with the clinical exam of superficial cellulitis with no evidence of abscess or necrotizing fasciitis. Although initial gram stain and cultures were negative, in the context of potential environmental wound contamination while carrying wood and possible inhibition of bacterial culture growth due to concurrent antibacterial therapy, the worsening leukocytosis raised concern for a superimposed bacterial cellulitis in this immunocompromised host, and antibacterial therapy was continued with ampicillin/sulbactam that was later changed to amoxicillin/clavulanate upon discharge.

**Figure 2:**
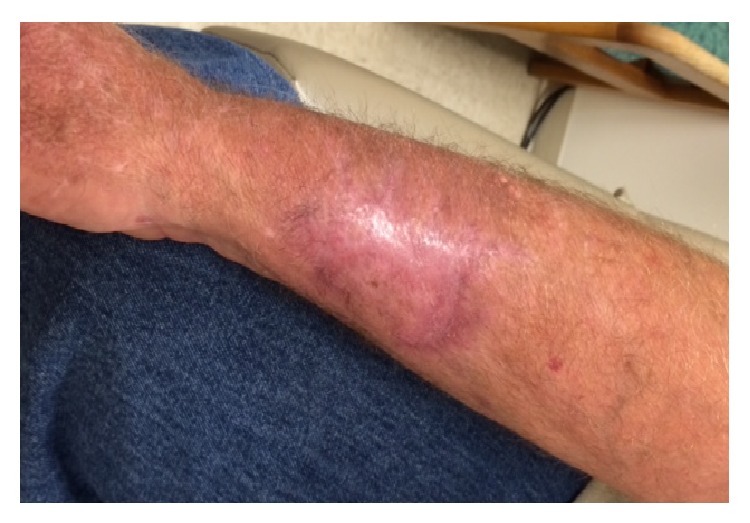
Healed right forearm lesion 6 months after symptom onset and at the conclusion of fluconazole therapy. The patient was seen in clinic 6 months after symptom onset and towards the end of the planned 6-month course of fluconazole therapy. Skin pigmentation changes remained, but the ulcer was entirely healed and there was no pain or evidence of subcutaneous involvement. Follow-up clinical evaluation was notable for the absence of systemic symptoms and serial serum cryptococcal antigen remained negative.

The patient's wound improved remarkably over the course of his hospital stay with near resolution of the surrounding erythema and edema (Figure 1(d)). He was sent home on oral fluconazole 8 mg/kg/day and a total of 14-day course amoxicillin/clavulanate. His prednisone was gradually weaned down to 10 mg/day; however, he had to increase the dosage to 30 mg/day due to asthma exacerbation after 2 months. At a one-week follow-up visit, his wound showed great improvement (Figure 1(e)). Within 3 months of fluconazole therapy, his wound showed complete healing. He tolerated fluconazole well and was continued on an 8 mg/kg/day regimen for an additional 3 months for a total duration of 6 months. He was last seen near completion of his fluconazole therapy (more than 6 months from symptom onset) without signs of relapse (Figure 2). Throughout this time he never experienced evidence of disseminated disease or meningeal involvement, and the serum cryptococcal antigen remained negative at the last clinic visit.

## 3. Discussion

Primary cutaneous cryptococcosis (PCC) is defined as identification of* Cryptococcus neoformans* in a skin lesion without evidence of simultaneous disseminated disease [35]. Most cases of PCC have been reported from Europe, Japan, and South America [6, 7, 9–12, 16–21, 30, 31, 35]. A nationwide survey conducted by the French Cryptococcosis Study Group identified 28 cases of PCC of the 1,974 total cryptococcosis cases reported to the National Reference Center for Mycoses from 1985 to 2000. Of the 28 patients with PCC, 50% were immunocompetent and only 11% had HIV infection, suggesting that PCC can develop regardless of immune status. Five patients were receiving long-term corticosteroid therapy [35]. Neuville et al. also observed that the skin lesions of PCC usually presented as solitary lesions resembling cellulitis, ulceration, or whitlow and were located on unclothed areas. In contrast, lesions from disseminated disease usually presented as scattered umbilicated papules resembling molluscum contagiosum [35]. Although the distinction is nonspecific and should not be used as a diagnostic tool, it is interesting to note that our patient's presentation is similar to many other patients with PCC. In addition, the French Cryptococcosis Study Group also highlighted that the spectrum of skin manifestations due to either disseminated or primary cutaneous cryptococcosis overlaps with other skin infections, necessitating biopsy for histopathological and microbiological diagnosis [35].

The treatment of choice for* C. neoformans* infection is determined by anatomic site of involvement and the host's immune status. While recent randomized-control trials are helping to clarify the evidence-based treatment of cryptococcal meningitis in patients with HIV/AIDS [36], management for unusual manifestations of disease in non-HIV-infected populations remains primarily guided by expert opinion. The Infectious Disease Society of America (IDSA) recommendation for patients with noncentral nervous system (CNS), nondisseminated* Cryptococcus* is oral azole therapy for 6–12 months (B-III recommendation) [37]. Furthermore, it is recommended that non-HIV-infected, nontransplant recipients be treated in the same fashion as those with CNS disease which consists of induction therapy with amphotericin B (or high-dose fluconazole if intolerant to amphotericin) plus flucytosine followed by fluconazole maintenance therapy [37]. Consensus on the duration of induction therapy in this population, however, is lacking as most data come from cohort studies of HIV-infected or organ transplant recipients [38]. The absence of well-controlled randomized trials for PCC or patients on chronic corticosteroids is a limitation in specific treatment guidelines. In most cases, disease remains limited to the skin, but there are reports of secondary systemic dissemination [39], including the CNS, and persistent antigenemia [40]. The patient in our case followed the typical pattern of uncomplicated PCC, manifesting neither systemic symptoms nor a positive serum cryptococcal antigen test.

To facilitate management in this case, we performed a literature search in PubMed using the search terms “cutaneous,* Cryptococcus*, and primary.” After excluding cases of documented or probable disseminated disease, we identified 43 reports in the English language from 1981 through 2014 (Table 1), 23 of which were reported over the last four years, although it is unclear if this is due to a true increase in PCC incidence or increased awareness among physicians, and thus reporting bias. Our search yielded a broad geographic distribution of reported cases among a wide age range in both immunocompetent and immunocompromised hosts. Compared with cases reported in immunocompromised hosts (44.1%), all of which were caused by* C. neoformans*, immunocompetent hosts had fewer reports of necrotic lesions and more commonly had infection with the emerging* C. gattii* species (23.3%). Interestingly,* C. gattii* was reported predominantly in Australia, Singapore, and Brazil, consistent with known restricted geographic distribution of* C. gattii* [41]. Other than three deaths, at least two of which were due to other causes, prognosis for PCC in these reports was overwhelmingly favorable as all patients were cured after as few as two weeks (in combination with surgical debridement) to up to ten months of antifungal therapy. In addition, fluconazole monotherapy has been increasingly reported in the recent medical literature, utilized in 17 out of 21 cases since 2011 and 5 out of 22 cases prior to 2011. For cases reported by the French Cryptococcosis Study Group, fluconazole was prescribed to 20 patients regardless of immune status for a median therapy duration of 32 days. Of these patients, 75% were definitively cured and 15% were attenuated [35].

Our patient had a favorable response to fluconazole monotherapy, amoxicillin-clavulanate, and topical wound care after only a few days of treatment. Regarding the use and role of antibacterial therapy in this case, the initial presentation suggested a worsening cellulitis despite adequate coverage for typical gram-positive bacterial causes of cellulitis (*Streptococcus pyogenes* and* Staphylococcus aureus*) initially with clindamycin and then with linezolid to treat possible methicillin-resistant* S. aureus* (MRSA). Failure to respond to these first-line empiric agents suggested either inadequate spectrum of antimicrobial activity due to an atypical or polymicrobial infection, presence or development of antimicrobial resistance in the pathogen, or need for drainage of an abscess not penetrated by the antimicrobial agent. Clinical worsening in this immunocompromised host with possible environmental inoculation while carrying wood thus prompted modifying coverage and bedside debridement to avert the rare, but potentially life-threatening circumstance of PCC with superimposed bacterial infection [42]. Piperacillin/tazobactam was initiated to cover gram-negative bacteria including* Pseudomonas aeruginosa* and anaerobes, and linezolid was changed to vancomycin for possible ongoing MRSA and other gram-positive activity. Either gram-negative organisms or a resistant* S. aureus* (including rare cases of linezolid-resistant* S. aureus*) from the purulent drainage would have been isolated in culture or visualized on gram stain. Fastidious pathogens and anaerobes, though difficult to isolate in the laboratory, would typically occur in the context of polymicrobial infection, and even if not isolated, evidence of bacteria would be expected on gram stain. The complete absence of microbiologic data to support a bacterial process in this case suggests that the antibacterials did not play a role in the healing process.

As this case illustrates, however, the overlapping manifestations between PCC and complicated bacterial skin and soft tissue infections may necessitate an early multidisciplinary approach involving infectious diseases and surgery consultants. Indeed, as our patient's treatment was complicated by intolerance to liposomal amphotericin B and a potential superimposed bacterial infection, we cannot dismiss that early debridement facilitated more rapid clinical improvement. Although data supporting the role of surgical debridement in the management of PCC is lacking [43], the use of this strategy as an augmentation to systemic antifungal therapy may be a consideration for patients in whom early drug intolerance is noted, or concerns of potential hepatotoxicity, QTc prolongation, or drug-drug interactions resultant from long-term azole therapy are substantial. Similarly, topical Pluronic antimicrobial cream for postdebridement wound healing is commonly used in our facility [44, 45], but the literature guiding its specific use in PCC is lacking. While the nystatin contained in Pluronic could be active against* Cryptococcus*, it should be emphasized that treatment of any cryptococcal infection should include systemic therapy, and the role of post-debridement topical therapy in this case was to facilitate wound healing in a patient taking chronic corticosteroids rather than for any direct anti-cryptococcal effect.

In summary, our case illustrates that prompt microbiologic testing and thorough evaluations for opportunistic atypical infections such as PCC should be considered in diabetic and immunocompromised patients who present with cellulitis that fails to respond to empiric antibiotic therapy. This case also demonstrates successful treatment of PCC with extended fluconazole monotherapy and local wound care. Clinicians should remain alert to the possibility of fungal skin soft tissue infections or coexistence of both bacterial and fungal infections. Although it is rare, coinfection of* C. neoformans* with bacterial infection can be devastating, particularly in immunocompromised hosts, if the diagnosis is delayed [42]. Finally, this case serves as a reminder to educate immunosuppressed patients about occupational risks from environmental exposures including unprotected handling of pigeons, decaying wood, and soil.

## Figures and Tables

**Table 1:** Clinical characteristics of cases reports of PCC in English language literature searched on PubMed.

Citation	Year	Age/gender	Region of origin	Immune status	Occupation/exposure	Site of lesion	Type of lesion	Species	Medical treatment	Duration of therapy	Topical care	Outcome
[6]	2014	68/male (M)	Southeast Brazil	Immunocompetent	Bus driver	Forearm	Nodule	*Cryptococcus gattii *	Fluconazole (200 mg/d)	40 days	None documented	Cured
[7]	2013	8/female (F)	Spain (child from Mongolia)	Immunocompetent	Not reported	Forearm	Macule	*C. laurentii *	Fluconazole (3 mg/kg/d)	2 weeks	None documented	Cured
[8]	2013	87/M	Rural area of Taiwan	Immunocompetent	Not reported	Arm	Indurated papules and plaques	*C. neoformans *	Fluconazole 400 mg/d for 2 weeks followed by 200 mg/d for 2 weeks	1 month	None documented	Cured
[9]	2012	66/M	Rural Central America	Immunocompetent	Not reported	Penis	Nodule	No fungal culture	Itraconazole (400 mg/d)	3 months	None documented	Cured
[10]	2012	89/M	Brazil	Immunocompetent	Collects firewood	Forearm	Ulceration	*C. gattii *	Itraconazole (400 mg/d)	3 months	None documented	Cured
[11]	2012	58/M	Greece	Immunocompetent	Poultry farmer	Hand	Ulceration	*C. neoformans *	Fluconazole (200 mg/d)	2 weeks	Surgical debridement	Cured
[12]	2012	Case series of 11 patients, mean age 71.2/9M2F	Brazil	5 immunocompetent, 6 on corticosteroid therapy	5 reported trauma or exposure to contaminated sources	Forearm	Circumscribed lesions ranged from an infiltrative plaque to a solid tumor mass	*3 C. neoformans* *4 C. gattii* *4 C. spp. *	Fluconazole (150 mg/d–400 mg/d)	1–6 months	None documented	9 cured, 1 unrelated death, 1 marked improvement
[13]	2012	55/M	Not reported	Renal transplant recipient	Not reported	Thigh	Umbilicated	*C. laurentii *	Amphotericin B + flucytosine	7 days (patient deceased)	None documented	Local lesion improving; however, patient deceased due to other causes
[14]	2011	67/M	Not reported	Immunocompetent	Poultry farmer	Forearm	Nodule	*C. neoformans *	Fluconazole (450 mg/d)	40 days	None documented	Cured
[15]	2011	37/M	Singapore	Immunocompetent	Forklift driver	Scalp	Nodule	*C. gattii *	Not reported			
[16]	2011	75/M	Brazil	Immunocompetent	Handles *Eucalyptus *logs	Forearm	Nodule	*C. gattii *	Fluconazole	5 months	None documented	Cured
[17]	2010	60/M	Slovenia	Renal transplant recipient	Cat scratch	Hand	Necrosis	*C. neoformans *	Amphotericin B + fluconazole	3 weeks (amp. + fluc.) + 15 weeks (fluc.)	Surgical debridement, amputation of the arm	Healed
[18]	2010	58/M	Italy	Immunocompetent	Collects firewood	Hand	Nodule	*C. neoformans *	Itraconazole (200 mg/d)	4 months	None documented	Cured
[19]	2005	43/M	Saudi Arabia	Immunocompetent	Driver for a furniture company	Forehead	Nodule	*C. neoformans *	Not reported			
[20]	2005	26/M	Japan	Long-term prednisolone therapy for minimal change nephrotic syndrome	Not reported	Leg	Nodule	*C. neoformans *	Intravenous fluconazole	3 months	None documented	Cured
[21]	2005	73/M	Spain	Liver transplant recipient	Insect bite in Costa Rica 2 days prior to presentation	Arm	Intense edema and suppuration	*C. neoformans *	Amphotericin B + fluconazole (100 mg/d)	15 days (amp. + fluc.) + 3 months (fluc.)	Surgical debridement and reconstruction	Cured
[22]	2004	57/M	Not reported	Lung transplant recipient	Gardener	Leg	Ulceration	*C. neoformans *	Fluconazole (200 mg/d)	2 months	None documented	Cured
[23]	2004	81/M	Not reported	Immunocompetent	Cattle farmer	Forearm	Pustules	*C. neoformans *	Fluconazole (400 mg/d)	2 months	None documented	Cured
[4]	2003	41/M	Southern Wisconsin	Immunocompetent	Puncture wound from hay bale wire in a barn	Hand	Erythematous nodule	*C. neoformans *	Fluconazole (400 mg/d)	8 weeks	Surgical excision	Cured
[24]	2002	46/M	Not reported	Immunocompetent	Not reported	Finger	Cellulitis	*C. neoformans *	Itraconazole (200 mg/d)	10 months	Surgical excision	Cured
[25]	2002	65/M	Brazil	Immunocompetent	Not reported	Forearm	Ulceration	*C. gattii *	Fluconazole (150 mg/d)	45 days	None documented	Cured
[26]	2000	36/F	Not reported	Liver transplant recipient for Budd-Chiari syndrome	Not reported	Leg	Ulceration	*C. neoformans *	Fluconazole (200 mg/d)	3 months	None documented	Cured
[27]	1997	75/M	Australia	Immunocompetent	Orchid grower	Forearm	Tender, erythema	*C. gattii *	Amphotericin B + 5-flucytosine Itraconazole (200 mg/d)	3 weeks 3 months	Normal saline and miconazole cream applied once daily	Cured
[28]	1997	77/M	Not reported	Long-term steroid treatment	Chronic obstructive pulmonary disease	Hand	Ulceration	*C. neoformans *	Amphotericin B + fluconazole	12 days + 6 weeks	None documented	Cured
[29]	1996	62/F	Not reported	Long-term steroid treatment	Sarcoidosis	Forearm	Bullous	*C. neoformans *	Itraconazole (400 mg/d)	Not reported	None documented	Cured
[30]	1995	73/F	Japan	Immunocompetent	Not reported	Cheeks	Ulceration	*C. neoformans *	No antifungal therapy	Not reported		Cured
[5]	1992	52/M	Not reported	Renal transplant recipient	Undergoing surgery for below knee amputation	Leg stump	Necrosis	*C. neoformans *	Amphotericin B	Patient deceased	Surgical debridement	Patient died of multisystem organ failure
[5]	1992	55/M	Not reported	Renal transplant recipient	Polycystic kidney disease	Forearm	Nodule	*C. neoformans *	Amphotericin B + 5-fluorocytosine	Not reported	None documented	Cured
[5]	1992	27/F	Not reported	Severe lymphopenia	Hereditary lymphangiectasia	Legs, lower abdomen, and groin	Necrosis	*C. neoformans *	Amphotericin B + 5-fluorocytosine	Not reported	None documented	Cured
[31]	1990	63/F	Japan	Immunocompetent	Housewife	Earlobe	Erosion	*C. neoformans *	Itraconazole (100 mg/d)	13 weeks		Cured
[32]	1986	7/M	Not reported	Nonspecific failure in cellular immunity	Not reported	Postauricular	Not reported	*C. neoformans *	No antifungal therapy			Spontaneous healing
[33]	1985	53/M	Not reported	Long-term corticosteroid therapy for asthma	Pigeon fancier	Wrist	Mass	*C. neoformans *	Ketoconazole	3 months		Cured
[34]	1981	81/M	Not reported	Immunocompetent	Not reported	Forearm	—	—	IV and oral miconazole	25 days	None documented	Cured

## Abbreviations

CSF: Cerebrospinal fluid
PCC: Primary cutaneous cryptococcosis.
